# Omics Data Integration Uncovers mRNA-miRNA Interaction Regions in Genes Associated with Chronic Venous Insufficiency

**DOI:** 10.3390/genes16010040

**Published:** 2024-12-31

**Authors:** Fatma Sarı-Tunel, Ayse Demirkan, Burcak Vural, Cenk Eray Yıldız, Evrim Komurcu-Bayrak

**Affiliations:** 1 Department of Genetics, Aziz Sancar Institute of Experimental Medicine, Istanbul University, 34093 Istanbul, Turkey; ftm.sari@hotmail.com (F.S.-T.); vburcak@istanbul.edu.tr (B.V.); 2 Graduate School Institute of Health Sciences, Istanbul University, 34093 Istanbul, Turkey; 3 Section of Statistical Multi-Omics, Department of Clinical and Experimental Medicine, School of Biosciences and Medicine and People-Centred AI Institute, University of Surrey, Guildford GU2 7XH, UK; 4 Department of Cardiovascular Surgery, Institute of Cardiology, Istanbul University-Cerrahpasa, 34098 Istanbul, Turkey; ceyildiz@hotmail.com; 5 Department of Medical Genetics, Istanbul Faculty of Medicine, Istanbul University, 34093 Istanbul, Turkey; ebayrak@istanbul.edu.tr

**Keywords:** chronic venous insufficiency (CVI), varicose veins (VVs), miRNA, mRNA-miRNA interaction

## Abstract

**Background/Objectives:** Chronic venous insufficiency (CVI), a chronic vascular dysfunction, is a common health problem that causes serious complications such as painful varicose veins and even skin ulcers. Identifying the underlying genetic and epigenetic factors is important for improving the quality of life of individuals with CVI. In the literature, many genes, variants, and miRNAs associated with CVI have been identified through genomic and transcriptomic studies. Despite molecular pathogenesis studies, how the genes associated with CVI are regulated by miRNAs and the effect of variants in binding regions on expression levels are still not fully understood. In this study, previously identified genes, variants, and miRNAs associated with CVI, common variants in the mRNA-miRNA binding regions, were investigated using in silico analyses. **Methods:** For this purpose, miRNA research tools, MBS (miRNA binding site) database, genome browsers, and the eQTL Calculator in the GTEx portal were used. **Results:** We identified SNVs associated with CVI that may play a direct role in the miRNA-mediated regulation of the *ZNF664*, *COL1A2*, *HFE*, *MDN*, *MTHFR*, *SRPX*, *TDRD5*, *TSPYL4*, *VEGFA*, and *APOE* genes. In addition, when the common SNVs in the mRNA binding region of 75 unique CVI related-miRNAs in five candidate genes associated with CVI were examined, seven miRNAs associated with the expression profiles of *ABCA1*, *PIEZO1*, and *CASZ1* genes were identified. **Conclusions:** In conclusion, the relationship between genetic markers identified in the literature that play a role in the pathogenesis of the CVI and the expression profiles was evaluated for the first time in the mRNA-miRNA interaction axis.

## 1. Introduction

miRNAs are approximately 21–23 nucleotide length single strand RNA biomolecules that do not code proteins but can regulate gene expression at the transcriptional and posttranscriptional levels. miRNAs can alter the stability or gene expression by binding to mRNAs [1,2]. miRNAs have been found to play a role in biological mechanisms and the pathogenesis of many diseases including chronic venous insufficiency (CVI) [3,4] and chronic venous diseases (CVD) [5,6]. There are studies showing miRNA expression changes in miRNA profiling studies conducted in varicose veins (VVs) [6,7]. It has also been shown that expression changes of some miRNAs play an important role in the pathophysiology of VVs and can be used for therapeutic purposes [6,7,8].

Vascular dysfunction, known as CVI, and is considered an important health problem that causes severe complications such as painful VVs and even skin ulcers at advanced stages [5,9]. Among patients over the age of 15 affected by lower extremity CVI, 15% are male and 25% are female [5,10]. There are studies showing that the risk rate increases significantly due to the influence of hereditary and environmental factors (body structure, pregnancy, gender, age) [3,4,11]. In the pathophysiology of CVI, many biological processes play a role, including venous valve insufficiency, venous hypertension, changes in the vascular wall, hypoxia, fibrin accumulation, the development of varicose veins, and inflammation [12,13]. The hypertension that develops in the venous system (increased hydrostatic pressure) obstructs the lymphatic drainage that should occur toward the venous system. This condition leads to the blockage of lymphatic channels due to increased inflammation. In patients, severe CVI insufficiency can result in the development of phlebolymphedema, a type of lymphedema [14]. The molecular aetiology of this progressive and recurrent dysfunction, which is very common and usually requires surgical intervention, is still not clearly known; however, an increasing number of studies implicate a role for miRNA-based gene expression regulation in the pathogenesis. miRNAs interact with mRNAs by binding to 3′-UTR (untranslated region), 5′-UTR, coding sequences (CDS), and promoter regions. After miRNAs are synthesized, they are secreted into extracellular fluids through exosomes and transferred to target cells or bound to proteins such as Argonaut proteins. Extracellular miRNAs may function as biomarkers for diseases or as signaling molecules in cell–cell interaction [15]. By the multivalent binding of additional regulators, such as GW182 proteins, to miRNA-AGO complexes, binding of more than one miRNA to the same mRNA can be achieved, thus, more than one miRNA may be involved in the regulation of a target mRNA [16]. While the binding of more than one miRNA to a single mRNA at close intervals is called “neighborhood” miRNA co-targeting [17,18,19,20], the other type of miRNA targeting, called “seed overlap” miRNA co-targeting [16], refers to the partial overlap of seed sequence regions of miRNAs binding to the same mRNA. As a result, miRNA-mRNA interactions are complex and determining these interactions experimentally in the laboratory is time consuming and costly. For these reasons, using various parameters that play a role in miRNA-mRNA interaction, complex computational methods have been developed for the identification of target mRNAs of miRNAs and made available for search through websites such as miRBase [21] (https://www.mirbase.org/, accessed on 8 October 2024 ), miRDB [22] (https://www.mirdb.org/, accessed on 23 December 2024), MirGeneDB [23] (https://www.mirgenedb.org/, accessed on 23 December 2024), MBS [24] (https://genome.ucsc.edu/s/corocla/MBS, accessed on 8 October 2024–10 November 2024), and miRWALK [25] (http://mirwalk.umm.uni-heidelberg.de, accessed on 8 October 2024–10 November 2024). Among them, the MBS [24] database, which is provided as an additional tool in genome browsers such as UCSC, combines tools that determine miRNA-mRNA binding sites using different algorithms such as Targetscan [26], PITA [2], and Miranda [27]. Among them, the miRTarBase is an important experimentally validated tool which is included for reference in miRWALK in silico tool [28]. The miRWALK uses the random-forest-based approach software TarPmiR, which is an miRNA-mRNA binding region prediction tool, and provides predictions by scanning the entire 5′-UTR, 3′-UTR, and CDS regions [25]. miRWALK [25] compares its data with tools such as Targetscan [26], Mirdb [22], and Mirtarbase [28]. These tools provide extensive opportunities for in silico study miRNAbased gene regulation networks.

In order to understand the structure, regulatory mechanisms, and functions of gene expression of non-coding RNAs, which are an important part of the human transcriptome, datasets have been created and search tools have been developed for bioinformatics ana-lyses [29,30,31], and developments in this area are still ongoing. This study is based on the hypothesis that SNVs located within mRNA-miRNA interaction regions may influence the regulation of gene expression, using data from genes, variants, and miRNAs associated with CVI identified through genomic and transcriptomic studies. To this end, the first step was to investigate whether the significant eQTL (expression quantitative trait loci) changes associated with common variants linked to CVI are located within the mRNA-miRNA interaction regions. Secondly, in the literature, the presence of miRNAs associated with CVI in the mRNA-miRNA interaction regions of the targeted genes (*ABCA1*, *PIEZO1*, *CASZ1*, *PTEN*, and *HAS2*) was investigated, focusing on SNVs that cause eQTL changes. In the second approach, we selected five target genes (*ABCA1*, *PIEZO1*, *CASZ1*, *PTEN*, and *HAS2*) according to the literature [21,32,33,34,35,36,37]. Our study aimed to investigate genes, genetic variants, and miRNAs associated with the pathogenesis of CVI, focusing on how variants may affect gene expression through mRNA-miRNA interactions via exploration using in silico tools.

## 2. Materials and Methods

**Figure 1:**
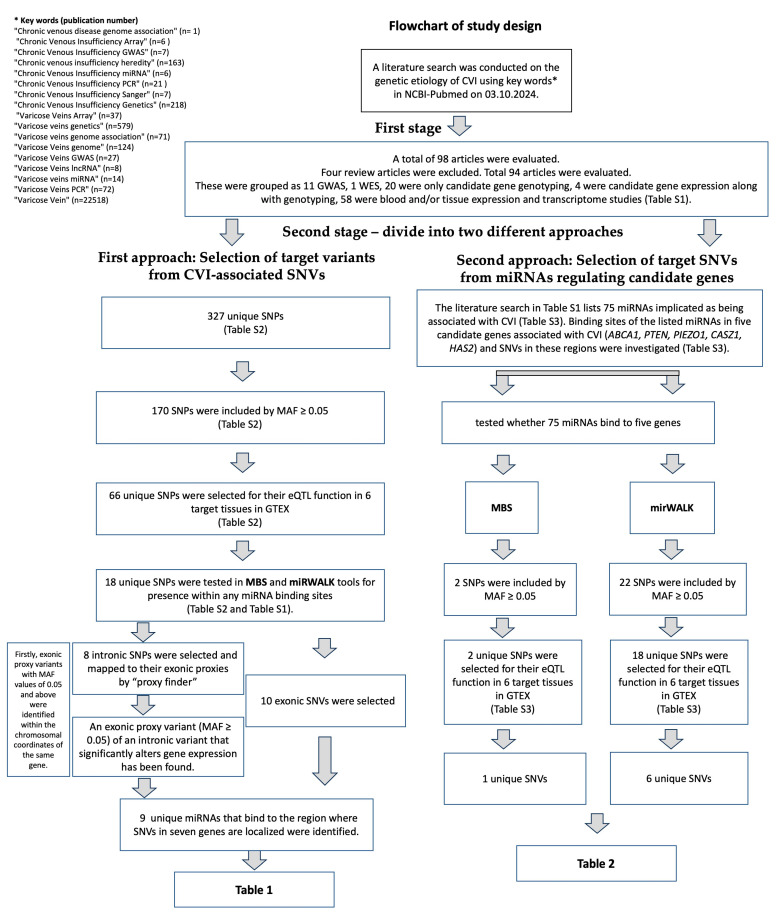
Workflow diagram showing the process of in silico investigation of variants that may be effective in CVI-associated mRNA-miRNA interaction.

The research flow of this study is summarized in Figure 1. According to this, in the first stage of this study, all genetic, epigenetic studies and reviews regarding CVI were examined using the PubMed (https://pubmed.ncbi.nlm.nih.gov, accessed on 3 October 2024) website. We screened all articles with search terms “Chronic venous disease genome association”, “Chronic Venous Insufficiency Array”, “Chronic Venous Insufficiency GWAS”, “Chronic venous insufficiency heredity”, “Chronic Venous Insufficiency miRNA”, “Chronic Venous Insufficiency PCR”, “Chronic Venous Insufficiency Sanger”, “Chronic Venous Insufficiency Genetics”, “Varicose Veins Array”, “Varicose veins genetics”, “Varicose veins genome association”, “Varicose Veins genome”, “Varicose Veins GWAS”, “Varicose Veins lncRNA”, “Varicose veins miRNA”, and “Varicose Veins PCR”, Varicose Vein” (Table S1). We excluded studies which are only studies on venous ulcers, studies on clinical treatment methods, and studies that have not been investigated or evaluated in terms of miRNA and genetic risk of varicose veins.

### 2.1. Establishing a List of CVI Risk SNVs and miRNAs

As a result of the CVI literature review, the general information of the studies (Sample Type, Research Type, Number of Cases/Controls, etc.), as well as the single nucleotide variants (SNVs) and miRNAs associated with the CVI, have been listed (Table S1).

### 2.2. Identifying SNVs Affecting Gene Expression in CVI-Associated miRNA Binding Sites

As a result of the literature review, 326 unique SNVs associated with CVI were listed (Table S2). Ensembl [38] (https://www.ensembl.org/index.html accessed on 23 December 2024) and Franklin (https://franklin.genoox.com accessed on 23 December 2024) databases were used for Minor Allele Frequency (MAF) of SNVs (Table S2). Franklin also provides MAF based on different populations. Each SNV’s MAF in global population in Ensembl (https://www.ensembl.org/index.html accessed on 23 December 2024) and Franklin (https://franklin.genoox.com/clinical-db/home accessed on 23 December 2024) databases was added to Table S2. Considering the prevalence of CVI, variants with an MAF value of 0.05 and above were filtered.

As specified under the heading “The effects of identified genetic determinants on gene expressions”, SNVs that significantly alter gene expression statistically are filtered.

We searched whether CVI risk SNVs (those with MAF ≥ 0.05 and *p* value < *p* value threshold) locate inside miRNA binding coordinates. First, we used MBS (miRNA binding site) database (https://genome.ucsc.edu/s/corocla/MBS accessed on 23 December 2024) to search for the binding site of the miRNAs by positional search [24]. miRNAs which positionally overlap with common SNVs are listed in Table S2. Second, we supported MBS results with miRWALK [25] (http://mirwalk.umm.uni-heidelberg.de accessed on 23 December 2024). We selected miRNAs that bind to the chromosomal coordinates in the location of SNVs and added them in Table S2**.**

The intronic SNVs associated with CVI in this list do not directly affect mRNA-miRNA interactions and are outside the scope of our study, so we included their common proxies (R^2^ ≥ 0.9, MAF ≥ 0.05) located in exonic regions within the same gene, using the LDproxy Tool (https://ldlink.nih.gov/ accessed on 23 December 2024).

### 2.3. Identification of SNVs Affecting miRNA Regulation of Known CVI Risk Genes

In this study, we searched whether known risk genes are then regulated by CVI-related miRNAs as a second approach. Our literature search uncovered 75 unique miRNAs associated with CVI. The *ABCA1*, *PIEZO1*, and *CASZ1* genes have been found to be associated with CVI in the literature [32,33,34,35,36]. According to the literature, the Hyaluronan synthase 2 (*HAS2*) gene, which was associated with varicose veins in the RNA sequencing study and was shown to be associated with varicose veins in the function study conducted in zebrafish, was selected as a candidate gene [37]. We also chose the *PTEN* gene, which has previously been studied in a limited number of studies on CVI, and it is involved in the MAPK and PI3K/AKt pathways, which are associated with the pathophysiology of CVI [21]. We tested whether our CVI risk genes (*ABCA1*, *PIEZO1*, *CASZ1*, *PTEN,* and *HAS2*) harbour binding sites for any of the 75 miRNAs and whether these binding sites harbour SNVs (MAF ≥ 0.05).

For this purpose, we gathered and integrated evidence in parallel from MBS and miRWALK, two algorithms which define miRNA targets using different approaches. We scanned these 75 miRNAs in the MBS tool, and checked whether they target the five risk genes (*ABCA1*, *PIEZO1*, *CASZ1*, *PTEN*, and *HAS2*) for CVI, and the chromosomal coordinates of binding sequences. Also, whether miRNAs have any binding sites in five candidate genes was tested using the miRWALK tool. Coding sequences were identified by using Ensembl (https://www.ensembl.org/index.html accessed on 23 December 2024) during the miRWALK routine. We identified whether the binding sites of miRNAs obtained from the miRWALK and MBS tools are located within chromosomal coordinates of risk genes and whether they contain common variants (MAF ≥ 0.05) using the NCBI variation viewer (https://www.ncbi.nlm.nih.gov/variation/view/ accessed on 23 December 2024) tool.

As the final step in this method, the research under the heading “The effects of identified genetic determinants on gene expressions” was conducted (Table S3).

### 2.4. The Effects of Identified Genetic Determinants on Gene Expressions

The remaining variant list after filtering variants with an MAF value of 0.05 and above (Tables S2 and S3), were tested for their eQTL effects by using “eQTL Calculator” in GTEx (https://gtexportal.org/home/ accessed on 23 December 2024) [15]. If they surpassed the experiment-wide *p*-value threshold set by the eQTL Calculator, the eQTLs were deemed statistically significant. We next filtered for tissue of interest; eQTL variants in genes expressed in at least one of six target tissues (Aorta, Coronary Artery, Tibial Artery, Cultured fibroblast cells, Skeletal Muscle, Whole Blood) were taken forward.

## 3. Results

### 3.1. Literature Research on CVI

In total, 98 articles were evaluated and four review articles were excluded, leaving us 94 articles to focus on (Table S1). Of the 94 articles, 11 were genome-wide association studies (GWAS), 1 was a whole-exome sequencing (WES) study, 20 were only candidate gene genotyping studies, and 4 were candidate gene expression along with genotyping studies. A total of 58 articles were blood and/or tissue expression and transcriptome studies. While six of the expression studies were only miRNA expression studies, five of them investigated miRNA and gene expression, and one of them studied miRNA and lncRNA and gene expressions. Finally, 45 articles focused only on gene expression, and the results were evaluated with further analysis (Tables S2 and S3). Finally, many studies have been conducted on varicose veins by different study designs, such as exome-wide associations, GWAS, family-based studies, proteomics, locus associations, and transcriptome analysis. The following subheadings briefly summarize the important results of studies with different study designs.

#### 3.1.1. The Transcriptome and Epigenetics Studies

Various hypotheses are being formulated on the pathophysiology of CVI, pointing to various pathways which we grouped into six, and we mention the most prominent ones from each group, while the full list is given in Table S1.

The first group includes studies on CVI pathology. Transcriptome analysis performed with RNA-Seq by Kuo et al. pointed to molecular changes in ATP production and use associated with VVs [39]. Studies have been published that attempt to unravel the pathways that play a role in the pathophysiology of varicose veins based on gene expression and protein studies. One of them is the PI3K/Akt/mTOR pathway which is assumed to be at the basis of vascular pathologies; PI3K/mTOR mRNA and protein levels were found to be significantly increased in CVI [40]. Another one is the HIF pathway which may be associated with various pathophysiological changes in the VV wall and that hypoxia may be a contributing feature to varicose vein pathogenesis. Expression of the prolyl-hydroxylase domain-2 (*PHD-2*) and *PHD-3*, *HIF-1alpha*, and *HIF-2beta*, which are HIF (hypoxia-inducible factors) regulatory enzymes, were found to be significantly upregulated in VVs compared to non-VVs. Increased expression was also detected at the protein level [9]. *ERK1/2* gene products are one of the enzymes of the MAPK pathway, an intracellular signal transduction system responsible for cell proliferation and survival, which is held responsible for CVI pathophysiology. In line with this, *ERK1/2* (*Mitogen-Activated Protein Kinase 3/1*) gene expressions were found to be higher in patients with CVI than in the control group, and *ERK1/2* gene expressions were found to be significantly higher in CVI patients with venous reflux than in CVI patients without venous reflux [41].

The second group involves the vascular development stages, specifically vasculogenesis and angiogenesis. These pathways include genes like *IGFBP6* (insulin-like growth factor binding protein 6) knockdown of which significantly attenuated cell proliferation and induced arrest in the S phase during the cell cycle occurs. Experiments showed that *IGFBP6* knockdown increased cyclin E ubiquitination, which reduced cyclin E expression and phosphorylation of *CDK2*. Furthermore, *IGFBP6* knockdown stopped centrosome replication, which subsequently affected vascular smooth muscle cells (VSMC) morphology. Findings confirmed that *IGFBP6* was involved in VSMC proliferation in varicose vein tissues [42]. Another interesting gene is the *HAS2* gene which was decreased in venous samples of patients with VVs. In addition, a zebrafish model with fluorescent vasculature and red blood cells was used to see the morphological changes of the venous system and blood flow. *HAS2* knockdown in zebrafish was found to result in dilated venous with static venous flow [37].

The third group consists of components that ensure vascular integrity, including ECM and basal membrane. Various studies have been conducted on the genes related to matrix metalloproteinases (MMPs), collagens, and tissue inhibitors of metalloproteinases (TIMPs). It is particularly noteworthy that genes that play a role in biological processes associated with varicose vein pathophysiology are studied in different populations with different study designs. Variable expressions of MMPs [43,44,45,46,47] and TIMPs [45,46,48] genes from extracellular matrix glycoproteins and variants in these genes have been investigated in venous tissues and blood samples in many studies. It was shown that at the gene expression and protein levels of *MMP1*, *MMP2*, *MMP9*, and inhibitors of these genes, *MMP1* increased in different levels of VVs and *TIMP1* increased only in advanced stages of the insufficiency, while no difference was found between MMP-1 and TIMP-1 in terms of total protein [49]. In the case–control study by Modaghegh et al., in which the effect of extracellular matrix change on varicose veins was investigated, it was found that the expression of the *MMP2* (metalloproteinase 2) gene increased and the amount of *MMP9* (metalloproteinase 9) decreased in the varicose group compared to the control group [44]. Genes known to be associated with extracellular matrix remodeling, including *COL3A1* (collagen III), *TIMP1* (tissue inhibitor of metalloproteinase I), *DPT* (dermatopontin), *MGP* (matrix Gla protein), and *TNC* (tenascin C) were determined to be overexpressed in VVs [48]. Collagen genes, in particular, *COL1A2* [8], *COL27A1*, *COL13A1* [32], *COL1A1* [50], and *COL3A1* [48], were found to show differential gene expression in VVs and some variants in the gene were found to increase risk of VVs.

The fourth group highlights important findings related to genes that may play a role in cell repair that could lead to the remodeling of vascular tissue. There are studies on expression changes in blood and vascular tissues and CVI-associated variants in some genes of the PDGF/VEGF growth factor family members that play a role in vascular development (*VEGFA* [47,51], *VEGF121* [52], *VEGF* [53]). In the study conducted by Hollingsworth et al., great saphenous vein samples were divided according to their anatomical positions and expression studies of the *VEGF* gene (soluble isoforms *VEGF121* and *VEGF165*) and receptors (*KDR*, flt-1 and soluble isoform of flt-1 s.flt-1) were performed. Varicose vein formation was correlated with increased expression of s.flt-1 and *VEGF121* when the sapheno-femoral junction was inadequate [52]. *VEGF* expression was also found to be significantly higher in patients in the VV group compared to the control group. It is predicted that it may play a role in the source of the pathology of CVI [53]. It has been shown in different studies that the *FOXC2* [54,55] gene shows differences in gene expression in blood and vascular tissues, and that some variants create a predisposition to varicose veins. While *FOXO3* (*FOXO3a*) and *APOE* genes were downregulated in VVs compared to the control group, *p53* gene expression increased significantly in the varicose vein group. In the same study, it was found that elastin and collagen accumulation increased in the VV group compared to the control group [44].

The fifth group includes genes involved in cell–cell interactions that are essential for maintaining the integrity of vascular tissue. The most prominent example is Piezo Type Mechanosensitive Ion Channel Component 1 (*PIEZO1*), which encodes a mechanically activated ion channel protein, in blood tissue, and in varicose veins, gene expression changes or CVI-associated variant studies have been associated with increased susceptibility to VVs [33,34,35,56].

The sixth group includes genes affecting blood stasis, which can have both mechanical effects due to fluid accumulation in the vascular system and biological effects such as viscosity and thrombosis, on which studies have also been published. Studies on the *CASZ1* gene, known to play a role in blood pressure, have found that there is a variant associated with the risk of VVs or a gene expression change in VVs [35,36,57]. Many studies on the *MTHFR* gene, which plays a role in vascular dysfunction mechanisms, have shown that variants increase the tendency towards VVs and that gene expression also changes in blood and vascular tissues [57,58,59].

A role for epigenetics highlights that since environmental factors (such as height, age, weight, etc.) play a role in the pathogenesis of CVI as much as genetic factors, it can be considered a multifactorial dysfunction. In fact, the treatment protocol often includes environmental factors like nutrition, exercise, and compression stockings. The significant role of environmental factors in the pathogenesis and treatment of the CVI highlights the need to investigate epigenetic mechanisms. In a study examining epigenetic changes in CVI patients from a different perspective, circRNAs involved in the regulation of microRNA-mediated gene expression were investigated by Zhang et al. In the study conducted in 2018, primary large saphenous vein varicosities (PGSVVs) using samples, 232 differentially expressed circ-RNAs were identified. In the computational pipeline analysis using the ten circRNAs whose expression changed most strikingly, it was predicted that it may affect miRNAs miR-103a-2-5p, miR-141-5p, miR-3692-5p, miR-4659a-3p, miR-4659b-3p, miR-4691-5p, miR-4778-3p, miR-6738-3p, miR-6792-3p, and miR-6873-3p [60].

In the transcriptome and DNA methylation analysis study conducted on varicose veins, 131 upregulated and 143 downregulated genes were identified, in addition to 459 hypermethylated and 119 hypomethylated CpG sites, concluding that *MFAP5* (Microfibril Associated Protein 5) acts as a master regulator of VVs morphology [61].

#### 3.1.2. Genome-Wide Association Studies

Until now, 11 GWAS studies have been performed on CVI. The most extensive among them utilized data from the UK Biobank, FinnGen Dataset, and a Russian biobank [35,56,57,62,63,64,65]. Intriguingly [62], the strongest associations in the three largest GWAS studies have been found in the *PIEZO1* and *CASZ1* genes. GWAS of the FinnGen dataset uncovered 50 genetic loci, 29 of which were new, including previously reported loci such as *PIEZO1*, *SOX9*, *ADAM15*, and *CASZ1*. These also include two X-chromosomal (*ARHGAP6* and *SRPX*) and two autosomal novel loci (*TGFB2* and *GJD3*) [63]. The GWAS of UK Biobank uncovered that 30 genetic loci were associated with VVs; the strongest associations found three SNPs in the *CASZ1*, *PIEZO1*, and 50 kB upstream to the *HFE* (hemochromatosis) genes [57]. In the GWAS study was established by Shadrina et al. integrating The Neale Lab (http://www.nealelab.is/ accessed on 23 December 2024) and the Gene ATLAS (http://geneatlas.roslin.ed.ac.uk/ accessed on 8 October 2024) UK Biobank data identified, 12 loci around genes *CASZ1*, *PIEZO1*, *PPP3R1*, *EBF1*, *STIM2*, *HFE*, *GATA2*, *NFATC2,* and *SOX*, contributing to 13% of the SNP-based heritability [35].

In a GWAS from a German population, the strongest associations with CVI were found for variants in the genes EFEMP1, KCNH8, and SKAP2 [62]. Additionally, in the largest GWAS conducted by Ahmed et al. in 2022, a study of 810,625 individuals found that more than 150 SNPs were associated with varicose veins [56]. Using UK Biobank and FinnGen GWAS datasets, a Mendelian randomization method revealed a causal association between serum plasma proteins IRF3, LUM, POSTN, RSPO3, and SARS and VV risk [66].

#### 3.1.3. Results of Articles on Candidate Gene Association Studies

Here, we briefly present the candidate gene strategy for confirmation purposes, as most of these genes and/or variants have already been covered by large TWAS and GWAS. Multiple case–control studies showed significant associations between VV risk and MMP9, TIMP2 (Chinese) [46], TIMP2 (Turkish) [67], HFE, VEGFA (Italian and Russian) [68,69] and VEGFA [51]. SNVs in MMP2 and MMP9 were also found to be associated with CVD [43]. In multivariate genotype analysis, it was found that two polymorphisms in the MTHFR gene previously associated with different dysfunction such as varicose veins and coronary artery in different articles may be involved in the morphological specification of primary varicose veins and contribute to the development of complicated CVI. It shows that the c.677C>T [70] variant of primary varicose veins is significantly associated with the trunk phenotype, whereas the c.1298A>C [71,72] variant is significantly associated with the perforator phenotype. When both c.677C>T and c.1298A>C variants exhibit a heterozygous genotype, patients are more likely to show both phenotypes. c.1298A>C variant was found to be strongly associated with congestive complication [59].

To summarize, our literature search including genome-wide association and candidate gene studies as well as gene expression profile studies pointed out 327 unique SNVs, 366 genes, and 75 unique miRNAs related to CVI, and those were followed up to obtain insights into the gene regulatory mechanisms involving miRNA pathways. All these data from the literature search of statistically significant CVI-associated genes, variants, and miRNAs are provided in Supplementary Table S1.

### 3.2. Bioinformatic Analyses

#### 3.2.1. In Silico Analysis of mRNA-miRNA Interactions of SNVs Associated with CVI

The obtained CVI-related literature on all CVI-associated genes, miRNAs, and SNVs contains 98 articles; the research is shown in Table S1. Using these data, we identified 327 unique SNVs associated with CVI and listed them in Table S2. After filtering them by a global MAF value of 0.05 and above in the Ensembl database, the remaining 170 SNVs were examined for eQTL functions in six target tissues in GTEx, and 66 unique SNVs were selected as most likely regulators of gene expression (Table S2). The co-localization of these 66 SNVs with any miRNA binding sites on gDNA was investigated in silico (Table S2). We determined that the expression profiles of 18 SNVs in 18 different genes determined by filtering according to the determined parameters were changed in the target six tissues and that they were regulated by 20 miRNAs (Table 1). Of these SNVs, 11 were in the exonic/UTR regions and 8 of them were in the intronic region (Table 1). After the target miRNAs were identified in the MBS database for each SNV, their positions in the transcript of the genes regulated by these miRNAs were confirmed with miRWALK. These eight intronic SNVs associated with CVI in GWAS and found to affect gene expression by in silico tools are listed in Table 1. However, miRNAs have been identified in the MBS database that may indirectly affect mRNA-miRNA binding by these SNVs that are not located in the gene transcript. While miRNAs corresponding to the genomic coordinate of the SNV are determined in the MBS database, when an intronic variant is examined, it is stated that the miRNA binds to an exon–exon junction in the mature mRNA. Therefore, miRNAs identified for intronic SNVs are located in the exon–exon junction region in the gene transcript. These deep intronic SNVs may have an impact on exon splicing or may be indirectly linked to another exonic SNV. Therefore, additionally, proxy variants in the encoded DNA regions of these seven intronic SNVs were considered. In six of the seven variants, no proxy variant with an MAF value of 0.05 or above was found within the same gene coordinates. One of the eight intronic variants (rs10007409) and a proxy variant (rs6833072) were found in the exonic region of the same gene (Table S4 and Table 1).

As a result of this approach, the variants rs1054852 (*ZNF664*), rs3917 (*COL1A2*), rs1799945 (*HFE*), rs3746106 (*MDN*), rs1801133 (*MTHFR*), rs35318931 (*SRPX*), rs61310274 (*TDRD5*), rs2232470 (*TSPYL4*), rs2010963 (*VEGFA*), and rs7412 (*APOE*) were identified as likely playing a direct role in mRNA-miRNA interaction. Of these, seven (hsa-miR-3678-3p for *HFE*, hsa-miR-3648 for *MIDN*, hsa-miR-4745-5p for *SRPX*, hsa-miR-6789-5p and hsa-miR-4688 for *TDRD5*, hsa-miR-3663-5p for *TSPYL4*, hsa-miR-210-5p for *VEGFA*, and hsa-miR-4755-3p and hsa-miR-5006-5p for *APOE*) were found to be associated with the same miRNAs in both the MBS and miRWALK tools.

#### 3.2.2. In Silico Analysis of mRNA-miRNA Interactions of Risk Genes Associated with CVI

In the second approach of our study, we investigated whether 75 miRNAs associated with CVI caused expression changes in five genes (*ABCA1*, *PIEZO1*, *CASZ1*, *PTEN*, and *HAS2*), which we selected as candidate genes for CVI according to the literature, by examining SNVs in mRNA-miRNA binding regions. For this, firstly using miRNA data from the literature in Table S1, 75 unique miRNAs associated with CVI are listed in Table S3. Using two different tools (miRWALK and MBS), it was investigated whether miRNAs found to be associated with CVI had SNVs in binding sites in the five candidate genes that we identified. SNVs that have an effect on gene expressions in blood and vascular tissues are listed in Table S3. And seven miRNAs compatible with the determined parameters were filtered and listed in Table 2. Of the five candidate genes we targeted among the seven miRNAs associated with CVI in the articles, it was determined that only three genes (*ABCA1*, *PIEZO1,* and *CASZ1*) had a significant effect on gene expression and common SNVs (MAF: 0.18–0.49).

There are no reported SNVs in or nearby the *ABCA1*, *PTEN*, and *HAS2* genes associated with CVI in the literature. Therefore, there are no variants in the *ABCA1*, *PTEN*, and *HAS2* gene in Table S2. Also, the rs11121615 variant in the *CASZ1* gene was found to be associated with CVD, but was not evaluated because it did not have a statistically significant eQTL effect. In Table S3, we show that only one unique variant of two different miRNAs associated with VV in the *CASZ1* gene had a statistically significant eQTL effect. In Table S3, 12 unique variants in the *PIEZO1* gene were found within the miRNA-mRNA interaction region, and only 5 unique variants in the *PIEZO1* gene were found to have a statistically significant eQTL effect. Although there are 150 variants in the *PIEZO1* gene associated with CVI in Table S2, all of them have been eliminated due to the determined filters (MAF and gene expression profiles). Considering these evaluations, no common variant was found when the variants in Table 1 and Table 2 were compared.

The workflow of the study is summarized in Figure 1. Among the numerous SNVs and miRNAs reported to be associated with CVI in the literature, statistically significant ones were investigated in this study using in silico tools. According to these analyses, in the first approach, 10 SNVs located in the direct mRNA-miRNA binding region that could affect the expression of the relevant gene, and in the second approach, 7 SNVs that were different from the others in the mRNA-miRNA binding region that could affect the expression of candidate three genes from CVI-associated miRNAs were detected (Tables S1–S4, Table 1 and Table 2).

## 4. Discussion

In this study, we investigated CVI-associated variants in the literature in terms of mRNA-miRNA interactions using in silico data and tools from two different perspectives. We identified that 18 variants associated with CVI in four GWAS [56,63,64,65] and five candidate genes studies [8,51,58,68,73] altered gene expression and were located within mRNA-miRNA binding regions in the first approach. As a second approach, we identified the presence of seven polymorphic variants that change gene expression in the mRNA binding regions of miRNAs associated with CVI in *ABCA1*, *PIEZO1,* and *CASZ1* genes. When comparing the findings from both methods, the miRNAs we identified, which bind to locations with SNVs associated with CVI, were found to overlap with the miRNAs associated with CVI in transcriptome studies from the literature. The common miRNAs are hsa-miR-216a-5p and hsa-miR-210-5p. However, when comparing the SNPs in the binding regions of miRNAs associated with CVI in risk genes to those CVI-associated SNPs identified in the literature, no common polymorphic variants were found.

In the first approach of this study, the region containing the rs2010963 variant in the *VEGFA* (Vascular Endothelial Growth Factor A) gene transcript has been shown to be bound to the hsa-miR-210-5p miRNA in MBS and miRWALK tools. In a study conducted by Shadrina and colleagues in the Russian population, it was found that the rs2010963 C allele reduces the risk of primary varicose veins [51]. In an miRNA profiling study conducted by Cui and colleagues on five great saphenous vein tissues, hsa-miR-210-5p was found to be downregulated [7]. The rs2010963 variant may have a protective effect by altering the mRNA-miRNA interaction and upregulating the regulation of hsa-miR-210. On the other hand, in the second method, which investigates common variants that alter the expression in binding regions of hsa-miR-210-3p miRNA on risk genes, we found the rs9928479 variant in the *PIEZO1* gene. The regulation of these variants, found in both the *VEGFA* gene, which plays a regulatory role in physiological and pathological angiogenesis through the proliferation and migration of vascular endothelial cells [5,51] and the *PIEZO1* gene, which is an ion channel protein that generates biological signals through mechanical activation, by the same miRNA, is highly significant. In the GWAS study, the miRNA (miR-216a-5p) binding to the region containing the rs9895127 [56] intronic variant in the *LINC01152* gene, which is associated with VVs, was found to be approximately eight times increased in varicose veins in the metabolic profiling study by Anwar and colleagues [21]. miR-216a plays a role in biological processes such as cell growth, proliferation, and autophagy through the activation of TGF and WNT signaling pathways [74]. The rs9895127 variant may affect the mRNA-miRNA binding energy, leading to a change in the gene expression of hsa-miR-216a-5p.

Based on the variants associated with CVI in the literature, we identified miRNAs interacting with variants in the 3′-UTR, 5′-UTR, CDS, and intronic regions. In a study by Jin Y. and colleagues, the rs3917 variant in the 3′-UTR region of the *COL1A2* gene was found to create a sequence that is complementary to a different miRNA (miR-382), which binds to the same region instead of the true miRNA. As a result, this led to the overexpression of the *COL1A2* gene. This situation has been suggested to be potentially related to varicose veins [8]. In our analysis, the chromosomal position of the rs3917 variant was identified as the seed region binding site for hsa-miR-3185. In the 5′-UTR region, the variants interacting with miRNAs include rs3746106 in the *MIDN* gene and rs1054852 in the *ZNF664* gene. It was found that different miRNAs bind to the position of the rs1054852 variant in both tools. In a GWAS conducted by Helkkula and colleagues, the rs3746106 variant in the MIDN gene was found to be associated with VV [63]. The interaction region of the miRNA (miR-3648) with rs3746106 was demonstrated in both tools. Overexpression of miR-3648 has been reported to increase cell proliferation via the WNT signaling pathway [75]. The rs3746106 variant may play a role in the cell proliferation stage of CVI pathogenesis by causing the overexpression of miR-3648. There is mRNA-miRNA interaction in the CDS regions of five variant genes associated with CVI. The rs1799945 variant in the *HFE* gene associated with CVI in the GWAS study [57] has been associated with an earlier onset of venous ulcers in a study by Zamboni and colleagues [69], while a study by Sokolova and colleagues [68] in the Russian population did not find an association with venous ulcers. Iron ions, which are a source of reactive oxygen species, can cause vascular damage in hypertension and cardiovascular diseases, potentially leading to CVI pathogenesis. The rs1799945 variant in the *HFE* gene, which is involved in iron ion metabolism, may affect miRNA interactions and alter gene expression, thereby contributing to the CVI pathogenesis. The rs1801133 variant in the *MTHFR* gene has been shown to be associated with CVI in studies conducted in different populations [58,59,71]. It is hypothesized that this association is due to a substitution of alanine with valine in a region presumed to be the catalytic domain of the MTHFR protein [76,77]. Interestingly, in our study, the rs1801133 variant was found to be located in the miRNA binding region. In mice with *SRPX* gene knockout, a pro-apoptotic effect has been shown [78]. Suppression of the expression of this gene may lead to increased angiogenesis, contributing to the CVI phenotype. miR-4745-5p binds to the CDS region of the SRPX gene, altering its expression, and this plays a role in CVI pathogenesis. This finding can be interpreted as a molecular correlate of the higher prevalence of CVI in women. In a study where the *TSPYL4* gene was silenced, WNT activity was found to increase by approximately 1.5 times [79]. When the pathways associated with the genes obtained from the GWAS study were analyzed, the non-canonical WNT signaling pathway was shown to be linked to CVI pathogenesis [56]. The rs2232470 variant in the *TSPYL4* gene, through its mRNA-miRNA (miR-3663-5p) interaction, may influence CVI pathogenesis by altering gene expression and activating the WNT signaling pathway. *APOE* is a well-known gene for dyslipidemia and Alzheimer’s disease. However, in 2024, Ismail et al. suggested that the rs7412 variant may be associated with venous insufficiency, but the relationship between it and cardiovascular diseases is complex [73]. These research data also draw attention to the effect of population-specific variability of allele frequencies and the presence of other comorbidities on genetic predisposition to complex dysfunction such as CVI.

In the second approach of the study, when we examined the interactions in CVI risk genes based on miRNAs associated with CVI, binding sites for three miRNAs were found in the ABCA1 gene. These binding sites include two common variants that alter gene expression. The rs2066714 variant is located in the binding sites of both hsa-miR-17-5p and hsa-miR-4778-3p. It was predicted that the presence of this variant could change the interaction between mRNA and miRNAs. It is interesting that no variant in the *ABCA1* gene has been reported to be associated with CVI in the literature articles and GWAS data we have reviewed so far. It is important to confirm this result in CVI patient samples. Also, we found that the four miRNAs associated with CVI have binding sites in the *PIEZO1* gene, and there are four common variants that alter expression at these binding localizations. This may provide insights into the genetic components of “phlebolymphedema”, which is linked to lymphatic dysfunction in CVI.

In this study, the integration of genomic, proteomic, and epidemiological data to delve deeper into the mRNA-miRNA interactions involved in the multifactorial mechanisms of CVI. Additionally, we attempted to gain more precise insights by analyzing mRNA-miRNA binding regions using two different tools that employ various algorithms. The fact that the obtained data could not be experimentally proven is an important limitation of this study. In GWAS studies, since we could not assess the mRNA-miRNA interactions of variants just below the *p*-value threshold, we cannot claim to have identified all the miRNAs that affect expression in CVI pathogenesis. Therefore, we were able to identify a limited number of miRNAs associated with CVI in the literature. The seven variants we found in the binding regions of CVI-associated miRNAs in risk genes may not have been associated with CVI in the literature because they fall below the defined threshold of *p*-value in this study. Furthermore, since intronic regions associated with CVI in the literature are not present in mRNAs, the interaction of intronic variants with miRNAs could not be evaluated with the current knowledge. For the seven intronic variants, proxy variants (R^2^ ≥ 0.90 and MAF ≥ 0.05) within the same gene were investigated. We found only the rs6833072 proxy variant in the *USP53* gene, which alters gene expression and is within an miRNA binding region. No analysis was conducted regarding whether the variants, whose effects on gene expression changes are unknown, are located in the miRNA binding regions. The determination of mRNA-miRNA binding regions is also affected by the limitations of in silico prediction tools used. Finally, this study focuses on SNVs in miRNA-binding regions that may play a role in the pathogenesis of CVI using in silico analyses. This study can guide future experimental studies.

## 5. Conclusions

In this study, for the first time, the relationship between CVI and genes or expression changes was examined from two different perspectives from the mRNA-miRNA interaction axis. As a result of in silico analysis of loci identified in GWAS studies related to CVI in the literature, variants in mRNA-miRNA binding regions that are consistent with CVI prevalence and affect gene expression were obtained with detailed filtering. According to the results we obtained from both approaches, the determination of different SNVs reveals the importance of perspective and the step-by-step effect of data analysis parameters. However, it is important to identify candidate genetic markers with high research potential among the numerous CVI-related genes, variants, and miRNA information in the literature. As a result, we believe that our findings will be important targets for further research.

## Figures and Tables

**Table 1:** The gene expression effect of SNVs associated with CVI in the literature and the investigation of miRNA binding sites with in silico analysis.

Reference	Variant ID	Gene ID	Coordinate	Location	MAF (Ensembl-Max Fr)	MAF (Franklin-Genoox Database)	eQTL Tissue	miRNAs (MBS)	miRNAs (miRWALK) (Confirmed)
[63]	rs5979390	*ARHGAP6*	X:11277189 C>T	Intron 1	0.32	Max: 29.0179% Aggregated: 19.2328% Türkiye: 24.8991%	Muscle—Skeletal	hsa-miR-1471	hsa-miR-1471 (CDS)
[64]	rs2057412	*CAB39L*	13:49408434 T>C	Intron 3	0.47	Max: 45.8002% Aggregated: 34.8988% Türkiye: 42.7555%	Muscle—Skeletal	hsa-miR-6772-3p hsa-miR-6791-3p hsa-miR-1910-5p hsa-miR-660-3p	hsa-miR-6772-3p (3′-UTR/5′-UTR) hsa-miR-6791-3p (3′-UTR/5′-UTR) hsa-miR-1910-5p (3′-UTR/5′-UTR) hsa-miR-660-3p (3′-UTR/5′-UTR)
[8]	**rs3917**	*COL1A2*	7:94431047 A>AGTTGTCC	3′-UTR (exon 52)	0.43	Max: 88.8176% Aggregated: 68.3878% Türkiye: 63.1477%	Cells—Cultured fibroblasts	hsa-miR-3185	The miRNA-mRNA interaction is not found in miRWALK
[68]	**rs1799945**	*HFE*	6:26090951 C>G	CDS (exon 2)	0.25	Max: 15.0159% Aggregated: 13.1952% Türkiye: 12.2731%	Artery—Aorta Artery—Coronary Artery—Tibial Muscle—Skeletal	hsa-miR-3678-3p *	hsa-miR-3678-3p * (CDS)
[63]	**rs3746106**	*MIDN*	19:1250110 C>A	5′-UTR (Exon 2)	0.49	Max: 54.4625% Aggregated: 35.6601% Türkiye: 46.0836%	Cells—Cultured fibroblasts	hsa-miR-3648 *	hsa-miR-3648 * (3′-UTR)
[58]	**rs1801133**	*MTHFR*	1:11796321 G>A	CDS (Exon 5)	0.50	Max: 47.7744% Aggregated: 31.8214% Türkiye: 29.9077%	Cells—Cultured fibroblasts Muscle—Skeletal Whole Blood	hsa-miR-6741-3p hsa-miR-10398-5p hsa-miR-3166	The miRNA-mRNA interaction is not found in miRWALK
[64]	rs10761247	*PHF2*	9:93641085 G>A	Intron 3	0.50	Max: 62.9801% Aggregated: 57.3044% Türkiye: 53.1736%	Artery—Coronary Cells—Cultured fibroblasts Whole Blood	hsa-miR-6846-5p * hsa-miR-328-5p * hsa-miR-328-5p	hsa-miR-6846-5p * (CDS) hsa-miR-328-5p * (3′-UTR)
[63]	**rs35318931**	*SRPX*	X:38149868 G>A	CDS (Exon 10)	0.10	Max: 8.4789% Aggregated: 7.0723% Türkiye: 2.4012%	Artery—Aorta	hsa-miR-4745-5p *	hsa-miR-4745-5p * (5′-UTR/CDS)
[65]	**rs61310274**	*TDRD5*	1:179593605 G>C	CDS (exon 3)	0.46	Max: 44.0589% Aggregated: 35.1162% Türkiye: 33.7299%	Artery—Aorta Artery—Coronary Artery—Tibial	hsa-miR-6789-5p * hsa-miR-4688 *	hsa-miR-6789-5p * (5′-UTR) hsa-miR-4688 * (CDS)
[65]	**rs2232470**	*TSPYL4*	6:116253920 C>A	CDS (exon 1)	0.37	Max: 99.844% Aggregated: 68.6071% Türkiye: 71.7184%	Artery—Aorta Artery—Tibial Cells—Cultured fibroblasts	hsa-miR-3663-5p *	hsa-miR-3663-5p * (CDS)
[51]	**rs2010963**	*VEGFA*	6:43770613 C>G	5′-UTR (exon 1)	0.45	Max: 76.5501% Aggregated: 68.3363% Türkiye: 59.9612%	Artery—Aorta	hsa-miR-210-5p *	hsa-miR-210-5p * (CDS)
[73]	**rs7412**	*APOE*	19:44908822 C>T	CDS (Exon 4)	0.17	Max: 10.5553% Aggregated: 7.424% Türkiye: 6.7358%	Whole Blood	hsa-miR-4755-3p * hsa-miR-5006-5p *	hsa-miR-4755-3p * (5′-UTR) hsa-miR-5006-5p * (CDS)
[56]	rs10007409 (proxy variant: rs6833072)	*USP53*	4:119221151 C>T	Intron 3 (proxy variant: Exon 1)	0.44	Max: 41.4991% Aggregated: 33.9762% Türkiye: 35.1876%	Whole Blood	hsa-miR-6818-3p	The miRNA-mRNA interaction is not found in miRWALK
[56]	rs11728719	*SORBS2*	4:185775018 A>C	Intron 2	0.24	Max: 24.5835% Aggregated: 17.5087% Türkiye: 15.912%	Cells—Cultured fibroblasts	hsa-miR-2276-5p hsa-miR-3194-5p hsa-miR-4692 hsa-miR-501-3p hsa-miR-10394-5p	The miRNA-mRNA interaction is not found in miRWALK
[56]	**rs1054852**	*ZNF664*	12:124011769 A>G	5′-UTR	0.50	Max: 57.4682% Aggregated: 39.0505% Türkiye: 40.2597%	Cells—Cultured fibroblasts Whole Blood	hsa-miR-661 hsa-miR-7107-5p	hsa-miR-3155a (5′-UTR) hsa-miR-1285-3p (5′-UTR) hsa-miR-3170 (5′-UTR)
[56]	rs41286076	*KLF5*	13:73060721 C>T	Intron 1	0.32	Max: 25.103% Aggregated: 17.5776% Türkiye: 25.9379%	Artery—Tibial	hsa-miR-1231	The miRNA-mRNA interaction is not found in miRWALK
[56]	rs9895127	*LINC01152*	17:72033667 T>G	Intron 1	0.49	Max: 64.1292% Aggregated: 56.086% Türkiye: 47.274%	Cells—Cultured fibroblasts	hsa-miR-216a-5p hsa-miR-4642	The miRNA-mRNA interaction is not found in miRWALK
[56]	rs76602912	*GNAS*	20:58884813 T>C	Intron 1/2	0.50	Max: 3.9459% Aggregated: 1.7677% Türkiye: 2.7167%	Artery—Aorta Artery—Tibial Cells—Cultured fibroblasts	hsa-miR-3065-3p hsa-miR-29a-3p hsa-miR-4767	The miRNA-mRNA interaction is not found in miRWALK

Legend: References, reference to the actual research article, Variant ID, RSid from dpSNP, Gene ID, ensemble gene ID, Coordinate genomics coordinate by Gh38, Location: critical location within the gene, miRNAs, miRNAs targeting the gDNA region according to MBS based on GhRC38. miRNAs (confirmed), targets confirmed by independent tool miRWALK based on GhRC38. Information such as gene chromosomal position and MAF value of variants associated with CVI were recorded. Variants with an MAF value of 0.05 and above and those that had a statistically significant effect on the expression change in the selected six tissues where the disease pathology was observed (*p* value > *p* value GTEx original threshold) were selected. The miRNA-mRNA interaction was evaluated by first searching in the MBS tool and then in the miRWALK tool for confirmation whether the variants affecting the expression change were in any miRNA binding region. Those with bold variant ID indicate those with a direct role in mRNA-miRNA interaction. *: Used to identify common miRNAs that regulate the same gene in both tools (MBS and miRWALK).

**Table 2:** Investigation of SNVs in chromosomal coordinates where miRNAs associated with SNVs in the literature interact with five candidate genes using in silico tools.

References	miRNA	Gene ID	Coordinate (Gh38)	MAF (Ensembl-Max Fr)	MAF (Franklin-Genoox Database)	eQTL Tissue	Variant ID	Variant Position
[6]	hsa-miR-183-5p	*PIEZO1*	16:88717330	0.18	Max: 95.8612% Aggregated: 89.8047% Türkiye: 89.1332%	Artery—Aorta Artery—Coronary Artery—Tibial Cells—Cultured fibroblasts Muscle—Skeletal Whole Blood	rs4782432 *	Exon
[21]	hsa-miR-135a-3p	*ABCA1*	9:104903754	0.31	Max: 22.124% Aggregated: 12.2863% Türkiye: 14.7477%	Muscle—Skeletal	rs1799777	5′-UTR
[21]	hsa-miR-136-5p	*CASZ1*	1:10649384	0.49	Max: 47.7473% Aggregated: 19.6429% Türkiye: 20.2619%	Whole Blood	rs284299	Exon
[60]	hsa-miR-4659b-3p	*PIEZO1*	16:88715442	0.19	Max: 15.6162% Aggregated: 12.0549% Türkiye: 4.4631%	Artery—Coronary Muscle—Skeletal	rs1061238	3′-UTR
[60]	hsa-miR-4778-3p hsa-miR-17-5p	*ABCA1*	9:104824472	0.49	Max: 66.6867% Aggregated: 16.7068% Türkiye: 21.5263%	Artery—Coronary	rs2066714	Exon
[7]	hsa-miR-127-3p	*PIEZO1*	16:88721296	0.26	Max: 90.9273% Aggregated: 87.3004% Türkiye: 83.4481%	Artery—Aorta Artery—Coronary Artery—Tibial Cells—Cultured fibroblasts Muscle—Skeletal Whole Blood	rs8043924	Exon
[7]	hsa-miR-210-3p	*PIEZO1*	16:88733714	0.29	Max: 18.3036% Aggregated: 1.0308% Türkiye: 0.0647%	Artery—Coronary	rs9928479	Exon

Legend: The target regions of miRNAs associated with CVI in five risk genes were identified using the MBS and miRWALK tools. References, reference to the actual research article, Variant ID, RSid from dpSNP, Gene ID, ensemble gene ID, Coordinate genomics coordinate by Gh38, Location: critical location within the gene, miRNAs, miRNAs targeting the gDNA region according to Information such as gene chromosomal position and MAF value of variants associated with CVI were recorded. Variants with an MAF value of 0.05 and above and those that had a statistically significant effect on the expression change in the selected six tissues where the disease pathology was observed (*p* value > *p* value GTEx original threshold) were selected. *: Used to identify SNVs at overlapping bases in the mRNA-miRNA binding region outside the “seed region”.

## Data Availability

All datasets analyzed or generated during the study are provided in the Supplementary Material.

## Supplementary Materials

The following supporting information can be downloaded at: https://www.mdpi.com/article/10.3390/genes16010040/s1, Table S1: The study designs of CVI-related articles and exploration of associations based on genes, miRNAs, and SNVs; Table S2: Especially in in vivo studies such as GWAS, the effect of SNVs associated with CVI on gene expression and the investigation of miRNA binding sites overlapping with these SNVs with in silico tools; Table S3: Investigation of SNVs located in localizations where miRNAs associated with CVI in the literature interact with candidate genes (*ABCA1*, *CASZ1*, *PIEZO1*, *PTEN*, *HAS2*) in in silico tools; Table S4: Investigation of exonic proxy variants of intronic variants in the miRNA binding region.
